# Correction: Li, Q.; Liang, S.Y. Incipient Fault Diagnosis of Rolling Bearings Based on Impulse-Step Impact Dictionary and Re-Weighted Minimizing Nonconvex Penalty Lq Regular Technique. *Entropy* 2017, *19*, 421

**DOI:** 10.3390/e22040483

**Published:** 2020-04-23

**Authors:** Qing Li, Steven Y. Liang

**Affiliations:** 1 College of Mechanical Engineering, Donghua University, Shanghai 201620, China; 2 George W. Woodruff School of Mechanical Engineering, Georgia Institute of Technology, Atlanta, GA 30332-0405, USA; steven.liang@me.gatech.edu

The authors were not aware of some errors and imprecise descriptions made in the proofreading phase, therefore, we wish to make the following corrections to this paper [1]: 

On page 3, Section 2, Paragraph 1, the sentence “The first impact approaches a step-like waveform and the second impact resembles an impulse-like waveform,” should be read as: “The first impact could be treated as step-like response (i.e., with low frequency components) and the second impact could be treated as impulse-like response (i.e., with high frequency components) [27].” 

On page 4, Section 2, Paragraph 5, the sentence “*τ* is system damping and *a* is the peak value ratio of impulse-like to the step-like impact” should be read as: “*τ* is system damping and *a* is the peak value ratio of the impulse-like response to the step-like response [27]”. 

On page 6, Section 3.2, the Paragraph 3 should be read as: “To overcome the above issue, inspired by the ideas of the unconstrained low-rank matrix recovery in Refs. [35–37] that many successful applications have implemented in the compressed sensing field [29–33], a new re-weighted minimizing nonconvex penalty Lq (0 < *q* ≤ 1) regular (R-WMNPLq) method is introduced, which is different from the ones studied in [35–37] where uniform random matrix (URM, i.e., the entries of matrix are random variables with uniform distribution) was used. In this work, the impulse-step impact dictionary is utilized for extracting the fault information from its observation or noisy data. The objective function is as follows:”.

On page 7, Table Algorithm 1, the title “Non-Convex-Penalty Smoothed Minimization Lq Algorithm (R-NSMLq)” should be read as “Re-weighted minimizing nonconvex penalty Lq regular (R-WMNPLq)“.

On page 7, Equation (12) should be read as $(D^{T}D+diag{\left(\frac{q\lambda }{{\left(\varepsilon_{k}^{2}+{\left\|\alpha^{\left(k\right)}[i]\right\|}_{2}^{2}\right)}^{1-\frac{q}{2}}}\right)}_{1\leq i\leq M})\alpha^{\left(k+1\right)}=D^{T}b$. 

On page 7, Equation (16) should be read as ${\left(\varepsilon_{k}^{2}+{\left\|x\right\|}_{2}^{2}\right)}^{1-\frac{q}{2}}{\left(\varepsilon_{k+1}^{2}+{\left\|y\right\|}_{2}^{2}\right)}^{\frac{q}{2}}\leq (1-\frac{q}{2})(\varepsilon_{k}^{2}+{\left\|x\right\|}_{2}^{2})+\frac{q}{2}(\varepsilon_{k+1}^{2}+{\left\|y\right\|}_{2}^{2})$. 

On page 7, the sentence “Theorem 1. Error estimation theorem” should be read as: “Theorem 1. Error estimation theorem [35,36]”. The sentence “To prove the theorem 1, the following two lemmas are required” should be read as: “To prove Theorem 1, the following two lemmas (i.e., Lemmas 1 and 2) [35,36] are required”. The citation of [35,36] after Lemma 1 (Page 7), Lemma 2 (Page 8) and Proof of Theorem 1 (page 17) should be cited accordingly. 

On page 9, Equation (23) should be read as $\frac{q}{2{\left(\varepsilon_{k}^{2}+{\left|x_{j}^{\left(k\right)}\right|}^{2}\right)}^{1-\frac{q}{2}}}\geq \frac{q}{2{\left(\varepsilon_{0}^{2}+\beta^{2}\right)}^{1-\frac{q}{2}}}$, and $\frac{1}{C_{4}}=\frac{q}{2{\left(\varepsilon_{0}^{2}+\beta^{2}\right)}^{1-\frac{q}{2}}}$. 

Due to the addition of new references, the order of the original citations has been changed correspondingly. 

The newly added references are as below:

27. Sawalhi, N.; Randall, R.B. Vibration response of spalled rolling element bearing: observations, simulations and signal processing techniques to track the spall size. *Mech. Syst. Signal Process.* **2011**, *25*, 846–870.
35. Lai, M.J; Wang, J.Y. An unconstrained *l_q_* minimization with 0 < *q* ≤ 1 for sparse solution of underdetermined linear systems. *SIAM J. Optim.* **2011**, *21*, 82–101.
36. Lai, M.J.; Xu, Y.Y.; Yin, W.T. Improved iteratively reweighted least squares for unconstrained smoothed *l_q_* minimization. *SIAM J. Numer. Anal.* **2013**, *51*, 927–957.
37. Wang, Y.; Wang, J.; Xu, Z. On recovery of block-sparse signals via mixed *l*_2_/*l_q_* (0 < *q* ≤ 1) norm minimization. *EURASIP J. Adv. Signal Process.* **2013**, *2013*, 76.

These changes do not influence the conclusions of this paper. The authors would like to apologize for any inconvenience caused. 
